# Editorial: Workflow optimisation for radiological imaging

**DOI:** 10.3389/fmed.2024.1487230

**Published:** 2024-10-10

**Authors:** Jie-Zhi Cheng, Minjeong Kim, Xin Yang

**Affiliations:** ^1^United Imaging Intelligence Co., Ltd., Shanghai, China; ^2^Department of Computer Science, University of North Carolina at Greensboro, Greensboro, NC, United States; ^3^School of Electronic Information and Communications, Huazhong University of Science and Technology, Wuhan, China

**Keywords:** artificial intelligence, radiological imaging, computer-aided diagnosis, cancer staging, metastasis prediction, quantitative analysis, prognosis prediction

Deep learning techniques have effectively addressed numerous technical challenges in medical image analysis, leading to the flourishing advancement of artificial intelligence (AI) applications in medicine. The scope of these applications may include the improvement of the image diagnostic workup, imaging workflow optimisation, burden alleviation of repetitive medical tasks, and comprehensive analysis for treatment planning. In recent years, numerous commercial AI applications have been introduced into clinical practice, demonstrating clear benefits. At the same time, there has been a proliferation of promising clinical validation studies of AI in the literature. Many of these important studies have demonstrated the efficacy of AI, supported by large datasets from multiple institutions and countries. To advance research on AI applications in radiology, we organised this Research Topic to explore new AI and machine learning methods for improving medical workflows, procedures, and diagnostic processes related to radiological imaging. We received numerous high-quality manuscripts, and after a rigorous review process, 16 of them were ultimately published. The published articles in this Research Topic can be categorised into several research topics of computer-aided diagnosis, cancer staging and metastasis prediction, quantitative analysis, and prognosis prediction. Each research topic is further summarised below.

## Computer-aided diagnosis

Li et al. utilised deep learning and radiomic techniques to diagnose Parkinson's disease based on T1-weighted magnetic resonance imaging. The VB-net demarcated the brain into 109 regions, from which a total of 2,264 radiomic features were extracted. The support vector machine was further applied to identify Parkinson's disease with high accuracy. Yang et al. focused on the early and rapid diagnosis of mild cognitive impairment, which is crucial for improving the prognosis of Alzheimer's disease. The structures of the bilateral hippocampus and parahippocampal gyrus were segmented from MR images for the Gaussian process models for the final identification of MCI. Wei et al. developed a novel prediction model that integrated multi-modal radiomic features derived from diffusion-weighted imaging and apparent diffusion coefficient images, along with multi-clinical features for the diagnosis of acute ischaemic stroke. Lin et al. proposed a deep learning-based diagnostic system to achieve early detection of esophageal cancer from non-contrast chest computed tomography (CT) images.

For differential diagnosis, Feng et al. extensively compared the performance of different machine learning approaches for the staging of testicular lesions in MR images. Lu et al. developed a deep learning radiomic model on multimodal images from ultrasonography, mammography, and MRI, which is further augmented with transfer learning, for malignancy identification of breast lesions. Cui et al. developed a CT-based radiomic model to distinguish between laryngeal squamous cell carcinoma and squamous cell hyperplasia. The applications of CAD are quite extensive, ranging from brain diseases to cancer problems in various parts of the body.

## Cancer staging, metastasis prediction

Wu et al. manifested the early stages of cervical cancer with radiomic features from the T2-weighted images and apparent diffusion coefficient maps. Tian et al. developed a segmentation-classification paradigm for rectal cancer (RC) lesions for the assessment of different T-stages.

For metastasis prediction, Ma et al. developed a clinical-radiomic nomogram, which leveraged deep learning techniques, preoperative MR images, and clinical characteristics, to achieve accurate prediction of lymph node metastasis in RC. Fu et al. developed multitask prediction models for High-grade serous ovarian cancer with radiomic profiling on preoperative CT scans. Specifically, the radiomic features were derived from manual ROIs, and then classification models were constructed with the feature selection scheme to predict the assessments of R0 resection, lymph node invasion, and distant metastasis status. Cancer staging and metastasis prediction are very useful for treatment planning.

## Quantitative analysis

Sun et al. employed ResUNet to delineate the testis from the MR scans for the measurement of testicular volume, which is essential for evaluating function and pathology. Jiang et al. utilised radiomic analysis on dual-energy CT images of the pancreas to establish a quantitative biomarker for type 2 diabetes mellitus. Deep learning algorithms were applied to segment the pancreas and were sequenced with the later classifiers of random forest, support vector machine and logistic regression. Meng et al. combined the clinical features, CT imaging signs, and radiomic features for the differentiation of Mycoplasma pneumonia in adults and children. The radiomic features are well-correlated with CT imaging signs, which provided a representation of different CT imaging signs as potential quantitative biomarkers. Jia et al. found that peri-plaque pericoronary adipose tissue (PCAT) was more valuable than proximal PCAT for the evaluation of coronary atherosclerosis. Spectral parameters of peri-plaque PCAT could further improve diagnostic accuracy. The quantitative parameters of peri-plaque PCAT attenuation were shown to be potential biomarkers of plaque vulnerability and the haemodynamic characteristics of coronary atherosclerosis. The quantitative measurement can replace tedious manual drawing, while the biomarkers will be very beneficial for disease diagnosis and treatment decisions.

## Prognosis prediction

Xue et al. predicted the prognosis of early acute pancreatitis with a clinical radiomic model on CT scans. Prediction of disease prognosis is very helpful in the determination of treatment approaches.

AI applications in medicine are booming, and can potentially benefit various aspects of clinical practice, such as simplifying medical procedures, improving efficiency, increasing diagnostic accuracy, optimising workflow, etc. In this Research Topic, we have published articles on a wide range of applications like CAD, cancer staging and metastasis prediction, quantitative analysis, and prognosis prediction that used radiomics and CNN techniques to attain specific goals. In particular, the studies by Lu et al., Meng et al., and Xue et al. further applied the multimodal data for better performance. The integration of multimodal data may reflect the real practice of clinical diagnosis and may be the future research trend of medical AI. The recent progress of large model techniques may further resonate and expedite this research trend with a high capability of context and long-range reasoning.

In conclusion, We hope that the articles published in this Research Topic could serve as a reference for the promotion of more advanced AI studies in the medical field.

